# T28. QUALITY OF PARENTAL BONDING AMONG INDIVIDUALS WITH ULTRA-HIGH RISK OF PSYCHOSIS AND SCHIZOPHRENIA PATIENTS

**DOI:** 10.1093/schbul/sby016.304

**Published:** 2018-04-01

**Authors:** Oon Him Peh, Attilio Rapisarda, Kang Sim, Jimmy Lee

**Affiliations:** Institute of Mental Health

## Abstract

**Background:**

A child’s parental bonding, measured using the Parental Bonding Instrument (PBI), has been found to be associated with psychiatric illnesses. In particular, a significantly higher proportion of patients with schizophrenia tend to report affectionless-controlling mothers as compared to healthy controls.

This study aims to (i) investigate the applicability of the PBI tool in Singapore, using exploratory factor analysis, and (ii) explore the association between parental bonding, symptom severity and functioning across schizophrenia patients, individuals at ultra-high risk of psychosis (UHR), and healthy controls.

**Methods:**

Data from 59 schizophrenia patients, 164 UHR, and 510 healthy controls (N = 733) were collected. The Structured Clinical Interview for DSM-IV (SCID) was used to ascertain any psychiatric diagnoses. Positive and Negative Symptoms of Schizophrenia (PANSS) and Global Assessment of Functioning (GAF) were administered on UHR and patients. Social and Occupational Functioning Assessment Scale (SOFAS) was administered on HC and UHR. Calgary Depression Scale for Schizophrenia (CDSS) was administered on UHR only.

Two exploratory factor analyses of the PBI were conducted on maternal items and paternal items (oblimin rotation). PBI factor scores were calculated for each individual and compared across groups using one-way ANOVA. Multivariate backward regressions were conducted to elucidate the association(s) between parental bonding factors and the clinical scales.

**Results:**

Factor analyses revealed three-factor solutions for both maternal and paternal items, with factors ‘care’, ‘autonomy’, and ‘overprotection’. All the original ‘care’ items loaded onto the ‘care’ factor for maternal and paternal analyses. The original ‘control’ items were split into ‘autonomy’ (the degree to which children were allowed to make their own decisions, e.g. ‘gave me as much freedom as I wanted’) and ‘overprotection’ (e.g. ‘felt I could not look after myself’). Fit statistics suggested a good fit for both maternal items and paternal items (CFI > 0.9, TLI > 0.9). UHR were 1.61 times as likely to report affectionless-controlling mothers (OR = 1.61, 95% CI: 1.13–2.30, p = .008) and 0.52 times as likely to report having optimal mothers (OR = 0.52, 95% CI: 0.29–0.93, p = 0.028). No significant differences in paternal styles were reported.

Compared to HC, patients and UHR reported significantly lower maternal care (F(2,729) = 27.51, p < .001), higher maternal overprotection (F(2,729) = 17.00, p < .001) and paternal overprotection (F(2,711) = 9.30, p < .001) (bonferroni-corrected). Among UHR, higher paternal overprotection was significantly associated with higher total PANSS scores (β = .162, p = .045), higher PANSS general psychopathology subscores (β = .185, p = .022), lower GAF scores (β = -.188, p =.021), lower SOFAS scores (β = -.183, p = .024), and worse CDSS scores (β = .210, p = .009). Among patients, higher maternal overprotection (β = .444, p = .022) and paternal care (β = .400, p = .036) were associated with higher GAF functioning.

**Discussion:**

This psychometric investigation of the PBI among Asian participants yielded three-factor models, which deviate from the original two factors. Our findings replicate previous evidence of higher proportion of affectionless-controlling mothers among UHR and patients. Lower maternal care, lower maternal and paternal overprotection were reported. Paternal overprotection was associated with worse positive and negative symptoms of schizophrenia, worse social, occupational, and psychological functioning, and more severe depressive symptoms among UHR. Our results highlight the importance of addressing childhood parental bonding issues in early intervention services for UHR.

